# Feasibility of Endoscopy-Assisted Laparoscopic Full-Thickness Resection for Superficial Duodenal Neoplasms

**DOI:** 10.1155/2014/239627

**Published:** 2014-01-16

**Authors:** Ken Ohata, Masahiko Murakami, Kimiyasu Yamazaki, Kouichi Nonaka, Nobutsugu Misumi, Tomoaki Tashima, Yohei Minato, Meiko Shozushima, Takahiro Mitsui, Nobuyuki Matsuhashi, Kuangi Fu

**Affiliations:** ^1^Division of Gastroenterology, NTT Medical Center Tokyo, 5-9-22 Higashi-gotanda Shinagawa-ku, Tokyo 141-8625, Japan; ^2^Gastroenterological and General Surgery, Showa University Hospital, Japan; ^3^Department of Gastroenterology, The First Hospital of China Medical University, China

## Abstract

*Background*. Superficial duodenal neoplasms (SDNs) are a challenging target in the digestive tract. Surgical resection is invasive, and it is difficult to determine the site and extent of the lesion from outside the intestine and resect it locally. Endoscopic submucosal dissection (ESD) has scarcely been utilized in the treatment of duodenal tumors because of technical difficulties and possible delayed perforation due to the action of digestive juices. Thus, no standard treatments for SDNs have been established. To challenge this issue, we elaborated endoscopy-assisted laparoscopic full-thickness resection (EALFTR) and analyzed its feasibility and safety. *Methods*. Twenty-four SDNs in 22 consecutive patients treated by EALFTR between January 2011 and July 2012 were analyzed retrospectively. *Results*. All lesions were removed en bloc. The lateral and vertical margins of the specimens were negative for tumor cells in all cases. The mean sizes of the resected specimens and lesions were 28.9 mm (SD ± 10.5) and 13.3 mm (SD ± 11.6), respectively. The mean operation time and intraoperative estimated blood loss were 133 min (SD ± 45.2) and 16 ml (SD ± 21.1), respectively. Anastomotic leakage occurred in three patients (13.6%) postoperatively, but all were minor leakage and recovered conservatively. Anastomotic stenosis or bleeding did not occur. *Conclusions*. EALFTR can be a safe and minimally invasive treatment option for SDNs. However, the number of cases in this study was small, and further accumulations of cases and investigation are necessary.

## 1. Introductions

Endoscopic resection is a minimally invasive procedure for the treatment of superficial gastrointestinal neoplasms, and endoscopic submucosal dissection (ESD) has become widely used over the past several years [1–3]. Endoscopic resection of superficial duodenal neoplasms (SDNs), however, is practically very difficult and is occasionally accompanied by perforation because of its anatomical characteristics and the action of digestive juices [4–6]. The incidence of perforation induced by duodenal ESD was reported to be 21–35.7% [7, 8], being much higher than that (1.2–3.6%) induced by gastric ESD [9–11]. Moreover, the duodenal ESD procedure time for lesions with a mean diameter of 12.7 ± 14.8 mm was reported to be 89.1 ± 64.6 minutes, highlighting the difficulty of duodenal ESD.

On the other hand, surgical resection is invasive. In addition, it is difficult to determine the site and extent of the tumor from outside the duodenal lumen in laparoscopic surgery [12, 13]. Insufficient resection will result in tumor recurrence, and too large resection may lead to postoperative duodenal stenosis.

To overcome these problems, a new procedure called laparoscopy-assisted endoscopic full-thickness resection (LAEFR) using ESD technique has been proposed and reported in two case reports [14, 15]. This procedure involves several steps including endoscopic observation of the tumor within the duodenal lumen, marking around it, incising the peritumoral mucosa and submucosa by ESD technique using a needle knife, perforating the muscular layer in the incision line, and performing a full-thickness incision using an IT knife under laparoscopic supervision.

To reduce the risk of bleeding and shorten the duration of surgery in comparison with the procedure reported in the above mentioned case reports, we invented endoscopy-assisted laparoscopic full-thickness resection (EALFTR) technique for SDNs. The fundamental difference between the two procedures lies in whether full-thickness resection is performed using the ESD technique (LAFER) or laparoscopic surgery (EALFTR). The aim of this study was to investigate the feasibility and safety of a single center experience of FALFTR for SDNs.

## 2. Patients and Methods

Between January 2011 and July 2012, 22 patients (15 males and 7 females, mean age 65.4) with nonamupllary duodenal neoplasm without familial polyposis syndrome were included in the study. All the cases underwent EALFTR by an experienced surgeon (M.M) who had performed 1500 cases of laparoscopic surgery and an experienced endoscopist (K.O) who had performed 2000 cases of ESD. Characteristics of the patients are presented in Table 1. All data were collected and analyzed retrospectively. To establish the diagnosis and determine the indication, all the patients underwent preoperative endoscopy with biopsy. Before treatment, conventional endoscopy, including chromoendoscopy, was carried out, and the gross morphology, histological type of the lesions were examined according to the Japanese Classification of Gastric Carcinoma [16]. Lesions involving or adjacent to the ampulla, histologically proven adenocarcinoma, and lesions occupying over hemicircumeferential portion of the lumen were excluded from the indication. All patients also underwent computed tomography and abdominal ultrasonography before EALFTR. Cases with diagnosed carcinoma by the biopsy or lymph node metastasis were excluded from the indication.

Concerning the clinicopathological characteristics of the duodenal neoplasms, following data were collected: en bloc resection rate, R0 resection rate, resected specimen size, resected lesion size, operation time, estimated blood loss, number of port sites, rate of conversion to open surgery, complication (anastomotic leakage, stenosis and bleeding), time until restart of oral intake, and postoperative hospital stay length.

## 3. EALFTR Procedure for SDN

### 3.1. Setup for the EALFTR Procedure for SDN

**Figure 1:**
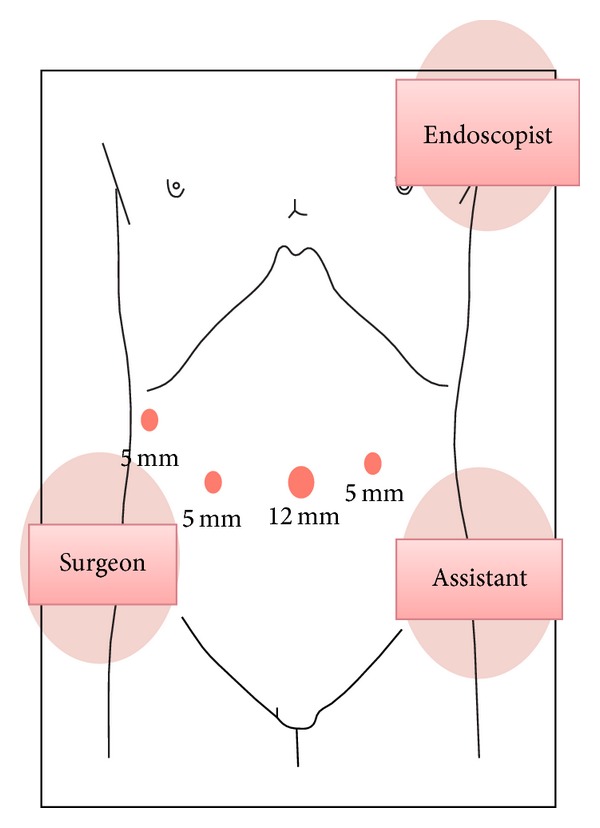
Setup for EALFTR. The surgeon stood on the patient's right side with the assistant and laparoscopist on the patient's left side and the endoscopic operator on the left side of the patient's head. EALFTR: endoscopy-assisted laparoscopic full-thickness resection.

All EALFTR procedures for SDNs were performed under general anesthesia and the patient was kept in supine position. The setup is shown in Figure 1. The surgeon stood on the patient's right side with the assistant and laparoscopist on the patient's left side and the endoscopic operator on the left side of the patient's head. To facilitate the laparoscopic procedure, a camera holder was inserted at the umbilical region using an open technique. Three additional 5 mm ports were inserted into right lower, right upper, and left lower quadrants, respectively, under laparoscopic view with pneumoperitoneum of 12 mmHg.

### 3.2. Mobilization of the Duodenum from the Retroperitoneum under Endoscopic Guidance

First, the jejunum was clamped with forceps at 5 to 10 cm distal to the Treitz ligament and the duodenum was mobilized from the retroperitoneum. Forward-viewing endoscope (GIF-Q260J; Olympus Medical Systems Corp., Tokyo, Japan) was inserted into the duodenum. While mobilization, in order to help the surgeon identify the tumor location, the endoscopist indicated the tumor location by endoscopically transmitted light guide and compressing at the duodenal wall by the tip of a catheter sheath. Blood vessels in the excision area around the tumor were dissected by the laparoscopist using a Harmonic ACE (Ethicon Endo-Surgery, LLC, Caguas, Puerto Rico 00725, USA).

### 3.3. Endoscopic Transmural Margination Around the Tumor

**Figure 2:**
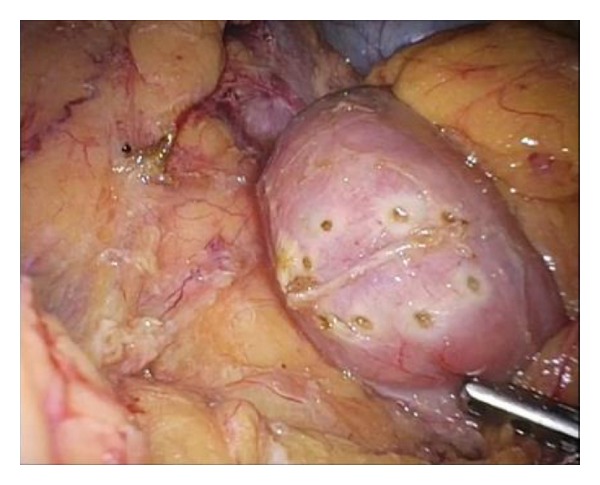
Laparoscopic view of intentional perforated peripheral marking. Under laparoscopic supervision, endoscopist penetrates the duodenal wall intentionally along the borderline of the lesion. Then, surgeon can identify the peripheral margin of the lesion under laparoscopic view.

Observing the border line of the SDN precisely, the endoscopist compresses at the margin of the lesion in the duodenal wall with the tip of a catheter sheath. Thus, the surgeon can recognize the tumor margin as exterior wall protrusion points in laparoscopic view. The laparoscopist secures enough space around the region of interest, and then the compression point is perforated by the endoscopist intentionally by sticking out the tip of the needle knife with the endo cut mode (VIO300D: ERBE Elektromedizin GmbH, Tubingen, Germany). After the tip of the needle has penetrated the wall, the endoscopist changes the generator mode to coagulation mode and coagulate the serosal layer, thereby putting a mark on the exterior wall which can be clearly recognized under laparoscopic view. By repeating this procedure, the peripheral margin of the lesion is marked around the tumor endoscopically (Figure 2).

### 3.4. Laparoscopic Sero-Muscular Layer Dissection and Closing the Defect

**Figure 3:**
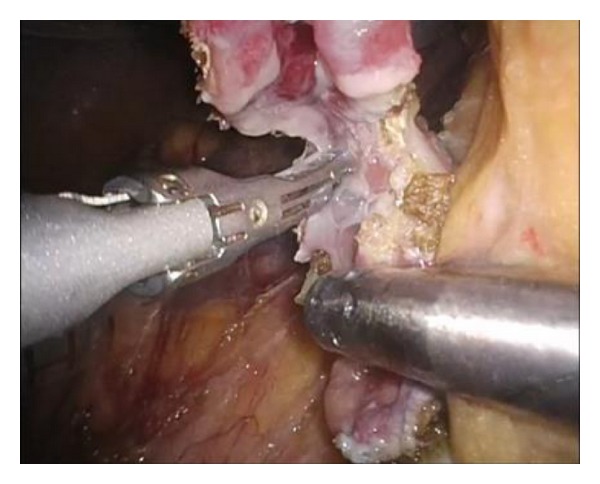
Laparoscopic view of full-thickness incision. After completion of entire circumferential seromuscular layer incision, the affected whole layer was dissected along sero-muscular layer incision circumferentially, using an ultrasonically activated device.

**Figure 4:**
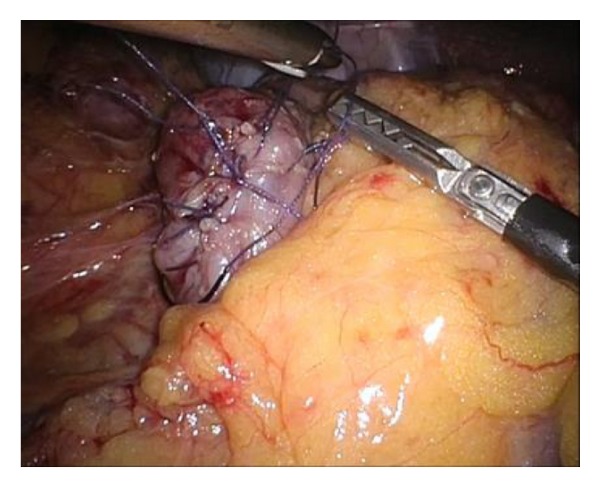
Laparoscopic view after the closure of the duodenal wall defect. The closure of the defect in the duodenal wall was performed by the laparoscopic hand-suturing technique.

The marking is identified under laparoscopy, and the sero-muscular layer is dissected laparoscopically along perforated markings circumferentially with an electrical scalpel. After entire circumferential sero-muscular layer incision, submucosa-mucosal layer is dissected along sero-muscular layer incision with ultrasonically activated device (Figure 3). The resected specimen is collected by a retrieval bag. The duodenal wall defect is closed by laparoscopic hand-suturing technique (Figure 4). After the operation, a drainage tube is placed beside the duodenum for a couple of days.

## 4. Histological Assessment

All resected specimens were flattened and fixed with thin needles onto corkboards before pathological fixation. The specimens were fixed by formalin. They were examined microscopically for histological type, depth on invasion, lateral resection margin, and vertical resection margin.

Neuroendocrine tumors (NETs) were diagnosed following the new histologic grading system of the 2010 World Health Organization classification for digestive system NETs [17, 18].

The rate of patients in whom the lesion was resected en bloc was defined as the en bloc resection rate, and resection en bloc with negative resection margins was defined as R0 resection.

Specialist GI pathologists reviewed all the specimens.

## 5. Result

Patient characteristics are shown in Table 1. Among the 22 patients, 15 were male and 7 were female, with a mean age of 65.4 ± 12.4 years. A total of 22 patients with 24 lesions underwent EALFTR. All the patients except for two had a single duodenal neoplasm, but one had double duodenal neuroendocrine tumors and another had double duodenal adenomas. As these lesions were located adjacent to each other in both cases, we removed them in a single piece. All lesions were found in screening endoscopy, and none of the patients had symptoms.

Clinicopathological characteristics of the duodenal neoplasm are indicated in Table 2. Six, sixteen, and two of the twenty four lesions were located in the 1st, 2nd, and 3rd portions of the duodenum, respectively. Of the 16 lesions located in the 2nd portion, 4 and 12 were present on the proximal and distal sides of the ampulla of Vater, respectively. Fifteen, six, and three lesions mainly occupied the anterior wall, posterior wall, and opposite side of ampulla of Vater. The circularity of 14 lesions was one forth or less and that of 10 lesions were one fourth to one half, and none was greater than one-half. In terms of tumor morphology, eighteen were elevated type and six were depressed. Of the 18 elevated-type lesions, 4 showed a submucosal tumor morphology and the remaining 14 were 0-IIa. The 6 depressed-type lesions were 0-IIc. Pathological finding of the specimens showed NET G1 (*n* = 4), adenoma (*n* = 13) and adenocarcinoma (*n* = 6). All 6 cases of adenocarcinoma were intramucosal carcinoma. Operative data in EALFTR are indicated in Table 3. All of the lesions were removed en bloc and in full-thickness, and the sizes of the resected specimens were as small as possible. The entire lateral and vertical margins of the specimens were negative for tumor cells. The mean sizes of the resected specimens and lesions were 28.9 mm (SD ± 10.5) and 13.3 mm (SD ± 11.6), respectively. The mean operation time and intra-operative estimated blood loss were 133 min (SD ± 45.2) and 16 mL (SD ± 21.1), respectively. Though EALFTR was started using four ports, some were added when necessary. The mean number of port sites was 4.2 (SD ± 0.8). None of the cases were converted to open surgery. The average time until the restart of oral intake was 7 (SD ± 4.4) days and the average period of the post-operative hospital stay was 15.1 (SD ± 7.7) days. EALFTR was performed without any intra-operative unfavorable events. Post-operative complications occurred in 5 patients: anastomotic leakage occurred in three patients (13.6%). The leakage was found in gastroduodenography, but none of them presented any symptoms. All leakages were minor and recovered conservatively.

The 3 cases with minor leakages were the 12th case in which the major axis of the resected specimen was 48 mm and the procedure time was 205 minutes, 19th case in which the major axis was 40 mm and the procedure time was 180 minutes, and 21st case in which the major axis was 45 mm and the procedure time was 207 minutes. The lesion was located on the anal side of the papilla in the 2nd portion in all 3 cases.

Duodenal hypoperistalsis occurred in two patients (9.1%). Both patients complained abdominal distention. Gastroduodenography showed no anastomotic stenosis, but the duodenal movement was decreased. We followed up several days conservatively, and the peristalsis gradually recovered and the patients could restart oral intake. Anastomotic stenosis and bleeding occurred in no cases. There was no procedure-related death.

## 6. Discussion

Duodenal neoplasms are rare and the standard treatment for nonampullary duodenal tumor has not been established [14, 15, 19, 20]. Local resection of nonampullary duodenal tumor using endoscopic submucosal dissection technique and laparoscopy has been described only in two case reports [14, 15]. This is the first report on a large number of cases of resection using combined endoscopy and laparoscopy.

With the aid of endoscopic light and intentional perforated marking guidance, the surgeon can determine the resection line precisely and accurately. Endoscopic circumferential incision for a duodenal lesion is extremely difficult technically and time consuming. The above mentioned previous case reports utilized circumferential incision method, but instead, we invented perforated marking. Sakon et al. [15] spent 156 and 179 minutes for resection, and Abe et al. [14] spent 200 minutes. Our mean procedure time was 130 minutes and apparently shorter than those previously reported cases. In fact, the procedure time in the first and the second case in our study was 110 and 140 min, respectively, which suggests that the time saving was not due to an accumulated experience. Since the laparoscopy and ESD operators were experts with abundant experience, as described above, the learning curve may not have had much influence. Perforated marking method is a comparatively easy and quick technique which would enable time saving. In addition, the laparoscopist cannot see intraluminal side incision. Our marks can be seen by the laparoscopist, because the coagulation markings involved the serosa. Direct visible margination enables the laparoscopist to decide precise incision line and it makes postsurgical duodenal wall defect as small as possible. Abe et al. [14] performed full-thickness resection endoscopically in the first part of the duodenum and they could resect the lesion precisely. Although it is not so difficult to operate the scope on the anterior wall of the duodenum, it is difficult to maneuver the endoscope on the posterior wall or in the third part of the duodenum. The indication would be strictly limited according to the location.

In addition, since our procedure involves an incision of the sero-muscular layer under laparoscopic visualization, it is easy to stop bleeding from blood vessels in the serosa, which may also lead to a shorter operation time.

One of the limitations of this procedure is that, if the target lesion involves the papilla or its vicinity, full-thickness resection is difficult to perform because the pancreas is located posteriorly.

In addition, if the duodenal wall defect after full-thickness resection is too large, postoperative duodenal stenosis may develop. The largest lesion in this study occupied 50% of the duodenal lumen and was successfully suture-closed laparoscopically without postoperative stenosis.

No major procedural complications occurred among the patients studied. Minor leakage occurred in 3 cases. These cases had common points: the resected specimen was large and the lesion was located on the anal side of the papilla in the 2nd portion. Although the number of cases was small and case accumulation is necessary, it is possible that minor leakage readily occurs when the resection range is wide or the lesion is located on the anal side of the papilla in the 2nd portion. Further investigation is necessary to consider the therapeutic indication. Two patients developed duodenal obstruction symptoms apparently due to excessive mobilization of the duodenum. To prevent this complication in the future, sacrifice of nerves and blood vessels should be strictly limited to the vicinity of the tumor. Excellently, en bloc resection was successfully performed in all patients. The possibility of intraabdominal dissemination of the tumor cells should be ruled out in a long time follow-up after surgery.

In conclusion, EALFTR enables successful en bloc, R0 resection, and full-thickness excision was achieved with an adequate surgical margin in all patients without severe complications. EALFTR can be a feasible, safe, and minimally invasive treatment option for superficial nonampullary duodenal tumors. However, the number of cases in this study was small, and further accumulations of cases and investigation are necessary.

## Figures and Tables

**Table 1:** Characteristics of the patients.

Patient, *n *		22
Lesion, *n *		24
Gender	Male/female	15/7
Age	Mean (years) ± SD	65.4 ± 12.4
Body mass index, (kg/m^2^)		22.7 ± 2.16
Preoperative complication, *n* (0)		
Symptom		
Nausea		0
No symptom		22

**Table 2:** Clinicopathological characteristics of duodenal neoplasms.

	Frequency, no. of cases
*Location in duodenum, portion (1st/2nd/3rd) *	
1st	6
2nd (proximal/distal to the papilla)	16 (4/12)
3rd	2
*Location in duodenum, dominant tumor-occupying direction (anterior wall/posterior wall/opposite side of ampulla of Vater) *	
Anterior wall	15
Posterior wall	6
Posterior wall/opposite side of ampulla of Vater	3
*Luminal circumference involved by tumor, % *	
<25	14
25–50	10
51–100	0
*Morphology, n *	
Elevated (submucosal tumor/0-IIa)	18 (4/14)
Depressed (0-IIc)	6
*Pathological features, n *	
NET G1	4
Adenoma	13
Adenocarcinoma	6

**Table 3:** Operative data for endoscopy-assisted laparoscopic full-thickness resection (EALFTR) for duodenal neoplasms.

		Frequency, no. of cases	%
En block resection, *n *		22	100
R0 resection, *n *		24	100
Resected specimen size, mm	Mean (mm) ± SD	28.9 ± 10.5	
Lesion size, mm	Mean (mm) ± SD	13.3 ± 11.6	
Operation time, min	Mean (min) ± SD	133 ± 45.2	
Intraoperative blood loss, mL	Mean (mL) ± SD	16 ± 21.1	
Number of port sites, *n *	Mean (*n*) ± SD	4.2 ± 0.8	
Conversion to open surgery, *n *		0	0
Postoperative meal start date, day	Mean (day) ± SD	7 ± 4.4	
Post-operative hospital stay period, day	Mean (day) ± SD	15.1 ± 7.7	
Post-operative complication, *n *			
Anastomotic leakage		3	13.6
Anastomotic stenosis		0	0
Anastomotic bleeding		0	0
Duodenal hypoperistalsis		2	9.1

## References

[1] Oyama T, Tomori A, Hotta K (2005). Endoscopic submucosal dissection of early esophageal cancer. *Clinical Gastroenterology and Hepatology*.

[2] Ono H (2005). Endoscopic submucosal dissection for early gastric cancer. *Chinese Journal of Digestive Diseases*.

[3] Saito Y, Uraoka T, Matsuda T (2007). Endoscopic treatment of large superficial colorectal tumors: a case series of 200 endoscopic submucosal dissections (with video){A figure is presented}. *Gastrointestinal Endoscopy*.

[4] Takahashi T, Ando T, Kabeshima Y (2009). Borderline cases between benignancy and malignancy of the duodenum diagnosed successfully by endoscopic submucosal dissection Endoscopic submucosal resection for duodenal tumor. *Scandinavian Journal of Gastroenterology*.

[5] Inoue T, Uedo N, Yamashina T (2013). Delayed perforation: a hazardous complication of endoscopic resection for non-ampullary duodenal neoplasm. *Digestive Endoscopy*.

[6] Fanning SB, Bourke MJ, Williams SJ, Chung A, Kariyawasam VC (2012). Giant laterally spreading tumors of the duodenum: endoscopic resection outcomes, limitations, and caveats. *Gastrointestinal Endoscopy*.

[7] Matsumoto S, Miyatani H, Yoshida Y (2013). Endoscopic submucosal dissection for duodenal tumors: a single-center experience. *Endoscopy*.

[8] Jung JH, Choi KD, Ahn JY (2013). Endoscopic submucosal dissection for sessile, nonampullary duodenal adenomas. *Endoscopy*.

[9] Abe N, Gotoda T, Hirasawa T (2012). Multicenter study of the long-term outcomes of endoscopic submucosal dissection for early gastric cancer in patients 80 years of age or older. *Gastric Cancer*.

[10] Chung I-K, Lee JH, Lee S-H (2009). Therapeutic outcomes in 1000 cases of endoscopic submucosal dissection for early gastric neoplasms: Korean ESD Study Group multicenter study. *Gastrointestinal Endoscopy*.

[11] Oda I, Saito D, Tada M (2006). A multicenter retrospective study of endoscopic resection for early gastric cancer. *Gastric Cancer*.

[12] Hoeppner J, Kulemann B, Marjanovic G, Bronsert P, Hopt UT (2013). Limited resection for duodenal gastrointestinal stromal tumors: surgical management and clinical outcome. *World Journal of Gastrointestinal Surgery*.

[13] Xie YB, Du J, Li Q (2012). Treatment and prognosis of patients with duodenal gastrointestinal stromal tumors. *Zhonghua Yi Xue Za Zhi*.

[14] Abe N, Takeuchi H, Shibuya M (2012). Successful treatment of duodenal carcinoid tumor by laparoscopy-assisted endoscopic full-thickness resection with lymphadenectomy. *Asian Journal of Endoscopic Surgery*.

[15] Sakon M, Takata M, Seki H, Hayashi K, Munakata Y, Tateiwa N (2010). A novel combined laparoscopic-endoscopic cooperative approach for duodenal lesions. *Journal of Laparoendoscopic and Advanced Surgical Techniques*.

[16] Sano T, Kodera Y (2011). Japanese classification of gastric carcinoma: 3rd English edition. *Gastric Cancer*.

[17] Tatsuta T, Yoshimura T, Hasui K (2013). Multiple gastric G1 neuroendocrine tumors with venous and lymphatic invasion. *Internal Medicine*.

[18] Klimstra DS, Modlin IR, Coppola D, Lloyd RV, Suster S (2010). The pathologic classification of neuroendocrine tumors: a review of nomenclature, grading, and staging systems. *Pancreas*.

[19] Okada K, Fujisaki J, Kasuga A (2011). Sporadic nonampullary duodenal adenoma in the natural history of duodenal cancer: a study of follow-up surveillance. *American Journal of Gastroenterology*.

[20] Abbass R, Rigaux J, Al-Kawas FH (2010). Nonampullary duodenal polyps: characteristics and endoscopic management. *Gastrointestinal Endoscopy*.

